# Cause and effect: the linkage between the health information seeking behavior and the online environment- a review

**Published:** 2014-09-25

**Authors:** R Bratucu, IR Gheorghe, RM Purcarea, CM Gheorghe, O Popa Velea, VL Purcarea

**Affiliations:** * “Carol Davila" University of Medicine and Pharmacy, Bucaharest, Romania; ** Dr. Carol Davila" Clinical Nephrology Hospital, Bucharest, Romania; *** National School of Political and Administrative Studies, Bucharest (SNSPA)

**Keywords:** Health information search behavior, seek online information, health care, health care services

## Abstract

Abstract

Today, health care consumers are taking more control over their health care problems, investing more time in finding and getting information as well as looking for proper methods in order to investigate more closely the health care information received from their physicians. Unfortunately, in health care consumers’ views, the trustworthiness of health authorities and institutions has declined in the last years. So, consumers have found a new solution to their health problems, that is, the Internet. Recently, studies revealed that consumers seeking for health information have more options to look for data in comparison to the methods used a few years ago. Therefore, due to the available technology, consumers have more outlets to search for information. For instance, the Internet is a source that has revolutionized the way consumers seek data due its customized methods of assessing both quantitative and qualitative information which may be achieved with minimal effort and low costs, offering at the same time, several advantages such as making the decision process more efficient.

 1. Services Marketing Concept 

 The diversity in the service sector is the consequence of several social factors which took place in the political, economic and technological environments. The main variables that led to the evolution of the tertiary sector were [**1**]:

 - The technological revolution encompassed two essential historical moments: the industrial era characterized by mass productions and productivity, emphasizing the delivery and the sales of material goods and, by the informational era, which referred to an evolution in the managerial communications through electronic resources. The technological revolution did not bring a raise in consumption but led to a higher rate of human resources. To solve this issue, people created the service sector. 

 - Globalization is another important factor in the evolution of integrating services. Actually, at the core level of globalization stood other 5 variables which contributed to its formation [**2**]: the market that was made out of global clients, the global distribution channels, competition which consisted of organizations that had headquarters on different continents, exporting or importing high standards applied to services, technology which offered the possibility to access telecommunications and online resources easily, government organizations such as commercial policy and environmental policy institutions, etc.

 - The social revolution was influenced by a new paradigm in human values. In this stage much emphasize was put on the qualitative aspect of services and not on the quantitative side. As the services sector begun to raise awareness, many experts from different fields such as economy, management and marketing defined the concept of services but without reaching a common accepted statement. 

 From the traditional marketing perspective, services represent a valuable surplus along with a good offered. As such, Kotler [**3**] considered services as part of the total offer of an organization, and consequently, he pointed out five category offers:

 - The offer made out of a pure tangible good which is not accompanied by a service

 - The offer which contained a tangible good but is accompanied by a service

 - The hybrid offer which comprised both tangible goods and services

 - The offer which consisted of a service accompanied by other goods and auxiliary services

 - The offer which is made out of only pure services. 

 2. The evolution of the services marketing

 Over the last decade, services marketing had had a very important part in developing the marketing research discipline. 

 Fisk et al [**4**] traced the evolution of services marketing in the scientific literature from the beginning in 1953 to its maturity phase in 1993. Within this process, they identified three stages of evolution: Crawling Out (1953-1979), Securring About (1980-1985) and the Walking Erect Stage (1986-1993), as shown in figure 1. 

**Fig. 1:**
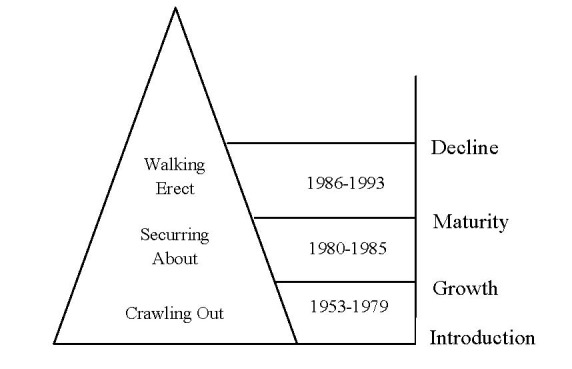
The evolution stages of Services Marketing

 In the Crawling Out stage, much was written on the services marketing regarding its existence as a field [**4**]. This stage begun in 1953, when articles on “how" and “why" services were different from goods. In this early stage, the identification of the distinctive characteristics of services was made. 

 The Securring About stage was the period when topics of the period when topics of the scientific articles shifted to distinguish between goods and services [**5**]. Lovelock [**6**] noticed that services marketing research was “emphasizing on drawing distinctions between goods marketing and services marketing and not enough on developing good insights for marketing practices in the service sector". As an answer he recommended several classifications of services in order to apply different marketing strategies. This was also the stage when the first papers appeared on service design and mapping [**7**] and service encounter [**8**].

 In the Walking Erect stage, services marketing became an established field in the marketing sector [**4**]. The topics of the publications evolved considerably switching to more subjects such as managing the quality service, designing and controlling service, managing supply and demand under constrained conditions [**4**]. This is also the period when methodologies to measure service quality were elaborated [**9**].

 3. Characteristics of services

 The four unique characteristics of pure services, namely, intangibility, heterogeneity, perishability and inseparability, are the ones that separate services from tangible goods. 

 Inseparability is the characteristic that refers to the fact that a producer or the provider of the service cannot be separated from the process of delivering the service at a certain moment in time. Inseparability occurs even if the producer is human or it’s a machine. In addition, services usually are first sold, then produced and consumed simultaneously [**10**]. Because the consumer has to be present when the service is produced and delivered, inseparability forces the client to get into “intimate" interaction with the producer. 

 Heterogeneity consists of a high variability in the performing activity of services. The process of delivering services can vary from producer to producer and from consumer to consumer.

 Perishability refers to the fact that services cannot be stored while intangibility is the key factor to differentiate goods from services. Kotler and Bloom [**11**] defined intangibility as the property of services that cannot be seen, felt, tasted, heard or smelled. Berry [**12**] described intangibility of being “easily defined, formulated or grasped mentally". Shostack [**7**] viewed intangibility as being impalpable and not corporeal. In other words, intangibility refers to the fact that services attributes cannot be perceived through the human senses. Furthermore, intangibility, in its first phase of research has been considered to be a single construct that was related to the lack of physical evidence [**13**]. In 1990, several scientists (e.g. [**14**]) proposed to divide intangibility in two dimensions: concreteness and specificity. Moreover, in 1998, Breivik et al [**15**] thought of splitting intangibility in other two dimensions: inaccessibility to senses and generality. Inaccessibility to the senses represented the lack of physical evidence, whereas generality referred to the capacity of a consumer not being able to identify precisely certain features, attributes or even definitions of a particular service. 

 Going further into the evolution of the concept, Laroche et al [**16**] adds a third dimension to the ones defined by [**15**], namely mental intangibility. Mental intangibility is the dimension in which consumers cannot imagine the service mentally because they lack the experience. Moreover, service intangibility has been thought to lower certainty of evaluation and increase the perceived risk [**13**]. Evaluation difficulty, according to [**13**], is a consumer’s perception which incorporates a difficulty to disseminate among alternatives in order to make a decision. Due to the fact that generality and mental intangibility are highly variable [**17**], they are expected to amplify the consumers’ perceptions about what the outcome might be. As a consequence, the evaluation process becomes more time consuming and effortful [**15**]. In this way, uncertainty is introduced leading to an increase in the difficulty of evaluation process. As such, given the process of uncertainty, subjects’ expectations of loss are growing considerably resulting in a high perceived risk. 

 Experts researched the conclusion that intangibility is positively correlated to the perception risk [**17**]. Actually, generality has been thought to increase the degree of perceived risk levels [**17**] because of the lack of specific attributes and at the high level of variability which leads to an increase of perceived risk (**Fig. 2**). 

**Fig. 2:**
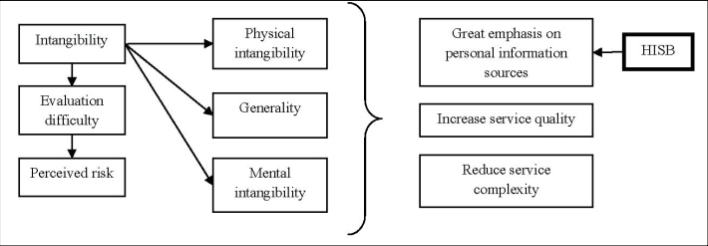
The link between intangibility, perceived risk and consequences

 3.1. Perceived risk

 Consumers’ perception of risk has been widely discussed in the past literature and has been shown to influence all purchase decisions to a certain degree. In consumer research [**19**], risk has been cited to represent the “consumers’ perceptions of uncertainty and adverse consequences of buying a product or service". A purchase decision involves risk when the consequences connected to it are uncertain and some results are desirable [**20**]. But if a situation has only one possible result which is surely a loss, it is not considered a risk because it lacks variability in results. Bauer [**20**] believed that perceived risk has two components: uncertainty as being the likelihood of unfavorable results and the consequences which were the importance of loss. The certainty of evaluation is linked to the consumers’ confidence in his/her ability to make a concrete purchase decision. Increased uncertainty has been associated with high levels of anxiety and discomfort [**21**].

 Kogan and Wallach [**22**] describe the concept of risk as consisting of two dimensions: the chance aspect where the focus is on probability and danger aspect where it is emphasized the severity of the negative consequences. Other researchers have divided the overall risk into multidimensional various risks or losses. As such five dimensions of risk have been identified as illustrated in fig. 3: performance, physical, financial, psychological, social risks, adding also time loss [**23**]. Performance risk is related to the consumers’ perception that a service purchased may not perform as desired and as a result, cannot deliver the perceived promised benefits. Physical risk refers to the overall threats of the appearance related to the quality of a service [**23**]. Financial risk is linked to concerns about the financial costs which include not only the actual costs but also the incidental costs such as travelling costs [**24**]. Psychological risk includes social risks described by friends’ and family’s knowledge and experiences with a service as being delivered inappropriate. Psychological risk relates also to the psychological aspect of being disappointed of not making the right choice [**25**]. Time risk is linked to the amount of time necessary to receive the service and the time loss as a result of service failure [**24**]. This component also incorporates travelling costs, waiting time, speed of service, convenience of opening hours. 

 The extent of the level of impact of perceived risk on the consumers’ behaviors depends on the importance of the goal, the penalty for not obtaining the goal and the amount of means committed to achieving the goal [**18**]. On one hand, when the perceived risk falls bellow an individual’s accepted value, it will be in great part ignored. On the other hand, an extreme high level of perceived risk can produce a postpone or even an avoidance of a service purchase. 

 In general, perceived risk is thought to be addressed during the early stages of consumer behavior [**27**]. Consumers try to evaluate virtually indistinguishable service alternatives. As a result, service consumers, in absence of information, rely heavily on personal recommendations and seek additional information about a service [**28**]. 

 This is a reality encountered in the health care sector. Nowadays, many consumers try to diminish the perceived risk of a health care service by searching for proper information. 

 4. Health care services 

 Health care is a service which most people need but do not want, in other words is an unwanted service not as the leisure services, for example. In addition, it can be considered highly troublesome but critically important [**29**]. It is the most customized service which consumers buy and yet its variability stands as proof for the difference between health care services and other services. For instance, in the health care sector, the supply of services increases demand but does not suggest profit, it rather means more physicians and hospital beds are required. 

 Berry and Beaudapudi [**29**] pointed out some important similarities and dissimilarities between health care and other services that are sometimes necessary to be known by marketing specialists in order to adopt the proper strategy.

 The similarities mentioned are the following:

 - The health care services are in essence intangible because a performance us actually being delivered during a health care consultation

 - Health care is a service which presents a considerable variability in physician performance and service delivery. The variability not only refers to changeable skills of physicians and service style but also in technical skills referring to the equipment used for analyses and diagnostic

 - Health care services are inseparable of the service provider mainly because it is a service that addresses the contribution of individuals. So patients for a clear, accurate diagnosis must be physically present where the health service is delivered. 

 - Similar to other services, health care is perishable. The health care organizations offer value through physician’s knowledge, equipment and time but when these elements are in their turn not created, the value cannot be measured.

 - Health care, similar to other services, is very technical and it requires a certain knowledge when it is used. Consumers not having the technical knowledge, transform health care in a credence service. 

 Although health care services are similar to other services in some characteristics, there are also certain dissimilarities which should be emphasized, as follows [**29**]:

 - Health care consumers are sick.

 - Health care consumers are reluctant;

 - Health care consumers relinquish privacy;

 - Health care consumers are at risk;

 - Health care providers are stressed. 

 In a nutshell, Thomas [**30**] indicated the unique characteristics of health care industry as follows:

 - On a market, the diffusion of information is usually spread equally between sellers and buyers. This is definitely not the case for health care consumers who lack the knowledge to evaluate a health care treatment.

 - Health care is not a field that is necessarily driven by financial purposes but rather by the well-being of patients.

 - A health care market is usually monopolistic, consumers not having much of a choice in finding a physician if they are insured.

 - In health care industry, the vast majority of consultations are made under ethical considerations.

 - In health care services, the payment of the service is made by a third party.

 - The health care industry is under the surveillance of the state even if it is fragmentized.

 5. The health care consumer information search behavior

 The consumer information search behavior has been analyzed from various perspectives, being developed actually on theoretical grounded foundations derived from Psychology, Sociology, Economics and Geography [**31**]. But the three disciplines that have focused more on consumer choice and information are Behavioral Science, Economics and Marketing.

 The Behavioral Science literature has concentrated on how individuals make choices based on conditions without taking into account the individuals’ characteristics and group membership. The conditions analyzed by the Behavioral Science include issues concerned with how much amount of information can affect the decision maker, in which way the certainty of the decision maker impacts the information search, and in which way can the variable time affect the decision maker. Nevertheless, behavioral science fails to give solutions on how information should be marketed to populations. 

 The Economic literature on consumer choice and information search introduces the concept of utility theory to explain behaviors. According to the economic literature, a consumer takes a decision based on the most beneficial and economic approach. Consequently, consumers are presumed to seek for information on the credence that information will improve their decision and will maximize their economic value decision. As such, they will search for information until they will reach the point when the cost for searching the information will be higher than the promised return he/she expects to get. In other words, the economic research proved that the consumer is going to search for information until the marginal benefits from doing so will equal the marginal costs [**32**]. According to Stigler’s theory [**33**], in the context of internet information search, the consumer will have more information at his/her disposal, making better costless decisions. Even if, Stigler’s theory [**33**] is thought to be normative, many researchers supported it with both analytical and empirical studies. The analytical studies as well as the empirical ones have refined Stigler’s initial theory [**33**], revealing other elements that may influence the information search behavior such as the amount of relevant information found, the prior knowledge consumers have before beginning a search, the attributes the consumers knew about the service/product before starting to search upon it, whether the recall of a certain product/service plays an important role in the searching process, etc. Some analytical findings have proved that consumers still have the tendency to search for purchase information a priori until they reach the point when their marginal costs have equaled their marginal benefits [**36**]. 

 The marketing literature has concentrated primarily, on the consumer’s buying process and identified several information regarding the needs of the specific target population groups. 

 Consumer information search behavior has in its center a construct which refers to the type of information searched. Therefore, the information search can be classified in internal and external [**34**] Internal information search takes place prior to the external one because it involves the consumer’s memory. Memory is considered, in many cases, to be the starting point in every information search, no matter if it is an external or an internal one. External information search was analyzed from various perspectives that focused on different methods that could develop and measure the consumers’ conscious efforts in minimizing uncertainty and risk while they are searching for several ways to acquire information with regard to a certain purchase. 

 After 1990s, in their quest to discover and develop the consumer behavior discipline, many researchers proposed models and theories for investigating the consumer search for information. In 1979, Bettman [**35**] introduced the theoretical information processing model. Bettman included and identified in his model several external and internal factors that could affect the consumer search behavior. The external factors are considered to be the environmental ones such as friends and family, the available time the consumer has to search for information and the necessary time for a consumer to make a choice. The internal factors are based on the individual’s characteristics such as information processing capacity, motivation, attention and, perceptual encoding, information acquisition and evaluation, decision processes and effects of learning. Bettman’s model, unfortunately was not pointing out the exact steps a consumer is likely to follow when searching for information [**36**]. 

 As a response to Bettman’s model, Howard [**36**] proposed another model called the consumer decision model that is composed of six interrelated elements: information, brand recognition, attitude, confidence, intention and purchase. 

 - The information constructs includes available facts about a product/service and the consumers’ percepts regarding that product/service, explaining to what extent the consumer absorbed the information about a product/service while being exposed to it.

 - Brand recognition refers to the fact that a consumer knows how to categorize the brand but has not enough knowledge to distinguish a certain brand from others

 - The attitude concept is considered by Howard [**36**] to be the fundamental construct in Social Sciences and signifies the level to which a brand satisfies a consumer’s needs.

 - Confidence is a consumer’s degree of certainty regarding an evaluation of a product/service when he/she tries to eliminate risk created by uncertainty.

 - Intention is the concept described by the consumers’ likelihood to buy a specific product/service which, in general terms, explains whether a consumer is going to buy or not the product/service.

 - Purchase is the stop point of the model when the money is exchanged for the product/service.

 With the introduction of the Internet in their search, consumers have changed their information search patterns. Internet is considered to be a limitless repository of information that can be available at all times from almost any part of the world [**37**]. Thus, internet has the capacity of storing vast amounts of information in different virtual locations. Furthermore, internet gives the opportunity to disseminate, organize, search, share information in an efficient and effective manner. But one of the most important characteristics of the internet is its interactivity, supporting several types of interaction from one-to-one, one-to-many, many-to-one, to many-to-many. These interactions can take place between humans, human-to-machine and between machines. 

 When consumers engage in information search on the internet, they do it with the help of browsers, search engines and other intelligent agents for acquiring information [**38**]. Browsers and search engines require a specific information before employment whereas the specific agents require specific inputs. There are several other reasons why consumers are using internet to search for health related information, as follows:

 - The internet makes a large volume and variety of information available with minimal resources of time, effort and money. The virtual environment of the internet is considered to be analogous with the real world in terms of physical appearance meaning that the information acquired from the physical real world source can be acquired in the same way as from the virtual world. For example, consumers can acquire information that is similar to the information they can get from traditional mass-media advertising, the same way as finding it directly from retailers’ or manufacturers’ websites or from word-of-mouth communications from family, friends, consumers or even experts via internet interfaces. Moreover, through internet, consumers can also obtain information from independent third-party providers, such as new media, university institutions, and nonprofit organizations. 

 - Consumers are looking online for health information because they can find data that was previously protected or unavailable.

 - The health-related information obtained by consumers has great value to them because it helps them understand better what to expect from the disease, the symptoms, treatments, etc. The internet provides the consumer what is called epistemic value [**39**]. 

 Getting a better insight into the health care information search behavior will offer researchers the possibility to discover how the process occurs. 

 The recent growth in consumer autonomy in health care and the urge to use media for health information gathering has led to an increasing interest in understanding the health information search concept. This recent interest in health information seeking has happened because of two different processes which took place almost simultaneously: the upsurge of the health care consumerism movement around the world [**40**] and the limitless access to health information available online. 

 Researchers as well as clinicians are interested in understanding some aspects regarding health care consumer behavior. They are interested in finding out how individuals obtain health information, where do they retrieve the information from, what types of information they prefer and how do they use the information sought. As a consequence, the health information seeking behavior (HISB) concept was introduced. According to Lenz [**41**], HISB is composed of a “series of interrelated behaviors that can vary along two main dimensions: extent and method". In other words, HISB include “actions that are used to obtain knowledge of a specific event or situation" [**42**] in a verbal or nonverbal behavior manner in order to “attain, clarify or confirm information" [**43**]. From a modern perspective, HISB is a “problem-focused coping strategy adopted by individuals to a threatening situation" [**44**] or is a strategy adopted by an individual when he/she feels the urge to comfort himself with the threatening situation by seeking more information about it [**44**]. In Czaja et al’s acceptance [**45**], HISB reflects the number of sources from whom an individual sought information from. On one hand, HISB is composed of two dimensions: the extent (the scope and the depth of the research) and the method (the type of information source used) [**41**]. On the other hand, HISB is suggested to consist of a cause (stress or threat) and a purpose (coping) constructs [**46**]. The information dimension describes the characteristics of the information sought, more precisely indicating the type and amount of information. The type of information refers to the content of the search. The amount describes how much health care information, one seeks and the level of the depth search. The method dimension focuses on the actions individuals use to obtain health-related information depending on the sources of information they use. Information seeking actions include direct or indirect questioning [**47**], as well as asking for clarifications, exchanging information with others, reading, browsing for information, listening and use of third parties’ information collections. 

 Nonetheless, HISB does not include the cases in which individuals are exposed to health-related information without a specific request from their side then, being the case of a passive receipt of information [**47**] or when the information is remembered. For example, if an individual is having a certain activity as watching television and he/she acquires information in a non-purpose manner, then this process is not considered to be a HISB [**47**]. However, passive acquisition of information can happen also during active information seeking [**46**]. Furthermore, Case [**48**] has used in his research study, the term information behavior to describe information seeking behaviors as well as unintentional or passive acquisition. Another case which might not be considered as HISB is the one when health professionals share information but without request [**43**].

## Conclusions

Seeking information makes individuals engage more in their medical decision making. The process itself helps consumers identify possible options, and at the same time, evaluate them, reduce uncertainty, doubt and encourage them to make a suitable decision. Actually, information seeking is identified as being a significant factor in an individual’s decision process that has an impact on the engagement in healthy lifestyles and preventive behaviors [**48**]. Although the information alone is not a proof that the individual has a healthy behavior, acquiring adequate information might motivate him/her to engage in healthy practices. Thus, HISB might influence an individual’s scope and nature of the information searched, on grounds such as judgments, beliefs and attitudes as well as on the number of alternative methods of action and knowledge against different risk perceptions and resources available. 

The health care consumer finds a true ally in the Internet because the health information is generally, easily organized by topic areas on health web sites and easily accessible whenever he/she needs a particular piece of information. Online health social networking sites can share the “wisdom of many". As such, health care social networking sites can shed light to personal stories, social norms and peer influence in order to help consumers understand the consequences of their behaviors on their health.
